# Machine learning for the prediction of minor amputation in University of Texas grade 3 diabetic foot ulcers

**DOI:** 10.1371/journal.pone.0278445

**Published:** 2022-12-06

**Authors:** Shiqi Wang, Jinwan Wang, Mark Xuefang Zhu, Qian Tan

**Affiliations:** 1 Department of Burns and Plastic Surgery, Affiliated Drum Tower Hospital, Medical School of Nanjing University, Nanjing, China; 2 School of Information Management, Nanjing University, Nanjing, China; Sejong University, REPUBLIC OF KOREA

## Abstract

Minor amputations are performed in a large proportion of patients with diabetic foot ulcers (DFU) and early identification of the outcome of minor amputations facilitates medical decision-making and ultimately reduces major amputations and deaths. However, there are currently no clinical predictive tools for minor amputations in patients with DFU. We aim to establish a predictive model based on machine learning to quickly identify patients requiring minor amputation among newly admitted patients with DFU. Overall, 362 cases with University of Texas grade (UT) 3 DFU were screened from tertiary care hospitals in East China. We utilized the synthetic minority oversampling strategy to compensate for the disparity in the initial dataset. A univariable analysis revealed nine variables to be included in the model: random blood glucose, years with diabetes, cardiovascular diseases, peripheral arterial diseases, DFU history, smoking history, albumin, creatinine, and C-reactive protein. Then, risk prediction models based on five machine learning algorithms: decision tree, random forest, logistic regression, support vector machine, and extreme gradient boosting (XGBoost) were independently developed with these variables. After evaluation, XGBoost earned the highest score (accuracy 0.814, precision 0.846, recall 0.767, F1-score 0.805, and AUC 0.881). For convenience, a web-based calculator based on our data and the XGBoost algorithm was established (https://dfuprediction.azurewebsites.net/). These findings imply that XGBoost can be used to develop a reliable prediction model for minor amputations in patients with UT3 DFU, and that our online calculator will make it easier for clinicians to assess the risk of minor amputations and make proactive decisions.

## Introduction

It is estimated that the number of diabetes patients worldwide will reach 300 million by 2025 [1]. Diabetic ulcers, particularly severe diabetic foot ulcers (DFU), are the most prevalent and severe diabetes consequence. When debridement and repair do not improve a patient’s diabetic foot condition [2], amputation becomes an unpleasant option. However, amputation can have a significant impact on the prognosis of individuals with DFU, decreasing their quality of life and increasing their chance of mortality [3–5]. According to statistics, the 5-year death rate for DFU patients will increase from 40% to 63% after amputation [1]. In addition, amputation multiplies the expense of treatment by a factor of five, hence increasing the load on the national health care system [6, 7].

DFU amputations can be divided into minor amputations below the ankle and major amputations above the ankle. Compared to major amputations as a life-saving alternative, minor amputations represent a larger clinical proportion (about 90%) of patients undergoing amputation [8]. Therefore, early diagnosis of the risk of small DFU amputation can benefit the majority of DFU patients, while early intervention for the risk of minor amputation is critical to prevent large amputations and preserve limbs [9, 10].

Machine learning is a subfield of artificial intelligence that has brought revolutionary changes to the field of health care through fast, efficient, accurate, and cost-effective computing decisions [11]. Machine learning plays an important role in the prediction of many common diseases, such as the diagnostic prediction of patients with type 2 diabetes [12] and the classification of cardiovascular diseases in patients with diabetes [13]. Several studies on DFU have shown the superior predictive performance of machine learning algorithms [14–19]. For the DFU diagnosis, Amith Khandakar et al. used thermogram images to establish a machine learning model based on CNN for early detection of DFU [15]. Rachita Nanda et al. constructed the prediction models for differentiating T2DM with DFU with four distinct machine learning methods [17]. For the DFU prognosis prediction, the Bayesian-based decision model [20] and the light gradient boosting machine model [19] were used to predict the amputation rate of DFU patients in different retrospective studies. Recently, a Chinese team focused on DFU mortality and amputation during the COVID-19 post-lockdown compared different machine-learning models [18]. However, these models’ performance was hampered due to a paucity of data. In a particular scenario, the choice of a machine learning algorithm depends on the sample dataset and the decision objective. The majority of studies utilized a single machine learning algorithm, they were unable to find the optimal algorithm.

In this study, we collected data on University of Texas grade 3 (UT3) DFU patients from two tertiary hospitals in eastern China. The University of Texas classification is a commonly used grading system, which is detailed in S1 Table. Patients with UT3 (bone or joint penetration) were selected because they constitute the majority of patients at tertiary hospitals and are the most susceptible to minor amputations. We used the following five supervised learning algorithms to filter out the best prediction model for minor amputation: logistic regression (LR), a long-standing statistical method to predict patients’ results based on the predictive variables of each patient [21]; random forest (RF), a mature learning algorithm widely used based on recursive methods [22]; decision tree (DT), a tree model in which each node has a question and each branch represents a result [23]; support vector machine (SVM), an excellent technology with independent integrity theory based on the optimal solution [24]; and extreme gradient boosting (XGBoost), an enhanced learning algorithm, which aims to transform weak learners into strong learners with high prediction accuracy [25].

Notably, the data collected by our team exhibits the class imbalance problem commonly associated with clinical data. To prevent its potential to lead to biased decision boundaries, we balanced the outcome variables using the synthetic minority oversampling technique (SMOTE) method, which has performed well in numerous studies [26–29]. To aid physicians, a final web-based calculator based on the best algorithm was also developed.

## Methods

**Fig 1:**
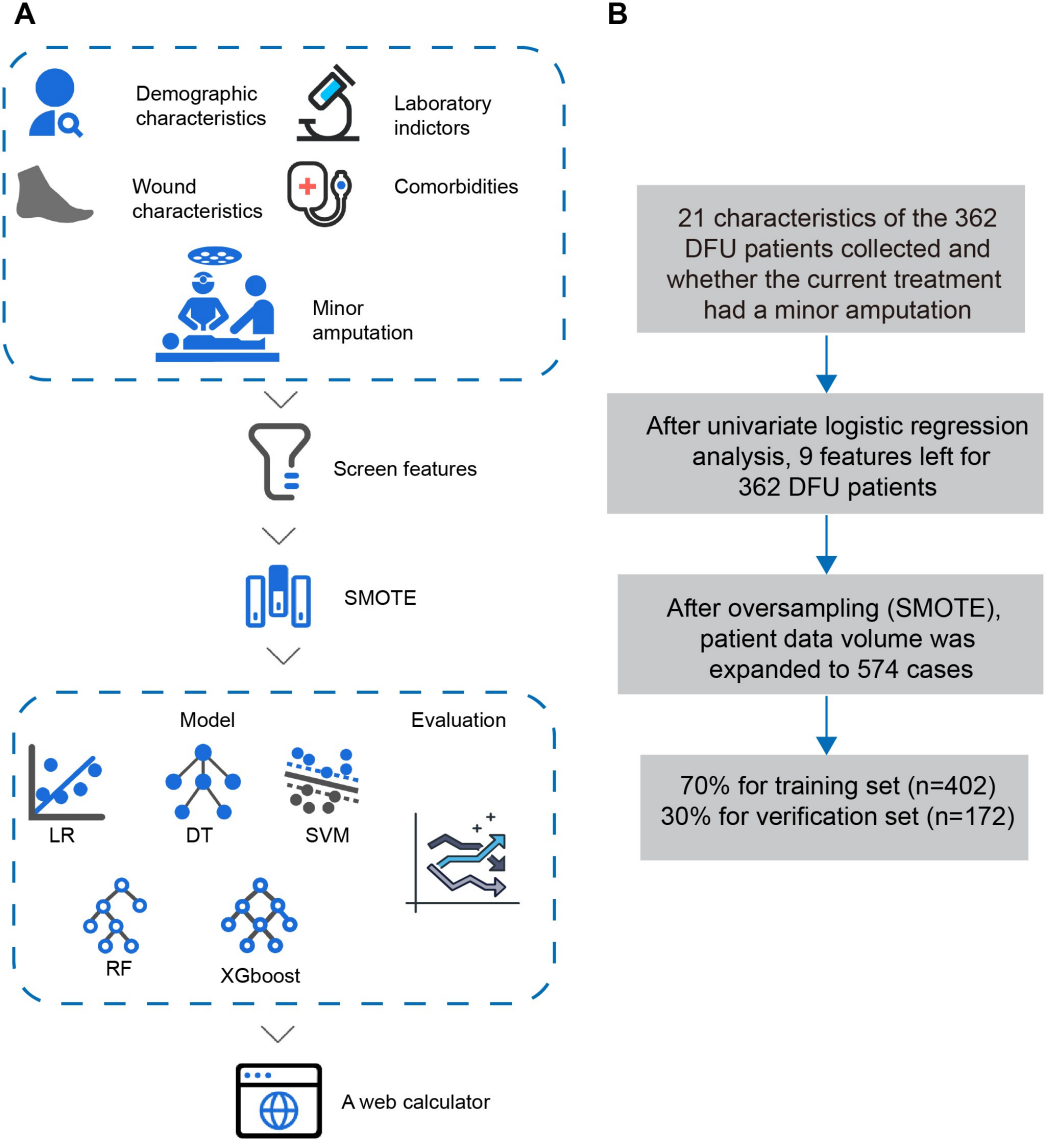
Study design flowchart and data screening flowchart. (A) Study design flowchart; (B) Data screening flowchart. ***Abbreviations***: DFU, diabetic foot ulcer; DT, decision tree; LR, logistic regression; RF, random forest; SMOTE, synthetic minority oversampling technique; SVM, support vector machine; XGBoost, extreme gradient boosting.

The study design flow chart and data screening flow sheet are shown in Fig 1. Below we present the study population, outcome and predictors, data processing, model building and model evaluation methods respectively.

### Study population

All clinical data were selected from *** Hospital and the *** Hospital from January 2018 to December 2019. Both hospitals treated DFU under multidisciplinary cooperation. Only patients with UT3 DFU were included in the study while patients who received major amputation, gave up treatment, or had incomplete information were excluded. The demographic data, wound characteristics and laboratory indicators of patients were collected from the medical records. According to the Helsinki Declaration, our study was approved by the Ethics Committee of Nanjing Drum Tower Hospital (No. 2020–10901). Informed consent of the participants was waived because of the retrospective study design and the use of anonymized clinical data.

### Outcome and predictors

#### Outcome

A minor amputation is defined as any amputation distal to the ankle joint.

#### Predictors

A total of 21 variables that have been shown to have a prognostic impact on diabetic ulcers in the previous literature were collected [30, 31]. All the enrolled predictors can be seen in the S2 Table. To avoid confusion, we have given specific explanations of the clinical indicators that may be controversial as follows:

Ulcer location: The location of the ulcer was judged by the endocrinologist and the burn plastic surgeon at the first visit to the hospital.
Wound duration: This period ranged from the first discovery to the first visit to the hospital.
Diabetic peripheral neuropathy (DPN): Clinical symptoms, such as finger/toe symmetrical sensory disturbance or abnormal nerve conduction velocity, are noted on electrophysiological nerve examination. Two of the following diagnostic criteria of DPN must be met [32]: (1) neuropathic pain, anesthesia, or other sensory abnormalities; (2) abnormal acupuncture sensation of the lower extremities or changes in the 10 g Sims-Weinstein monofilament test; or (3) decreased ankle reflex.
Smoking history: The definition refers to the standard recommended by the WHO in 1984, that is, smoking more than one cigarette a day for a continuous period of one month; otherwise, it is judged to be nonsmoking.
Drinking history: The criteria for being classified as a drinker includes a long history of drinking lasting for more than 5 years and daily alcohol consumption ≥80 g.
Hypertension: The criteria for determining hypertension are as follows: systolic blood pressure ≥140/90 mmHg or use of antihypertensive drugs with normal blood pressure.
Peripheral artery disease (PAD): If one or more lower extremity artery occlusions are found by Doppler ultrasound, it is diagnosed as peripheral artery disease [33].
Hyperlipidemia: Hyperlipidemia is known as lipid metabolism disorder and refers to total cholesterol, triglyceride, high-density lipoprotein cholesterol and low-density lipoprotein cholesterol exceeding the standard value.

To explore whether the variables were dose-dependent on the outcome of amputation of diabetic foot ulcers, we defined the abnormal values of variables according to clinical experience and guidelines [34–38]. Patients were divided based on 11.1–16.7 mmol/L and >16.7 mmol/L blood glucose. Patients were divided based on 25.0–34.9 g/L and <25.0 g serum albumin levels. Patients were divided based on 8–100 mg/L and >100 mg/L CRP levels. The duration of diabetes was divided into 0–10 years, 10–20 years and >20 years. Patients were divided based on creatine levels as follows: 134–186 μmol/L, 187–451 μmol/L, 452–771 μmol/L and >771 μmol/L. Details are provided in S2 Table.

### SMOTE for the imbalanced dataset

Our dataset consisted of 75 (20.7%) patients with minor amputation and 287 (79.3%) patients without any amputation. A class imbalance exists in the original dataset, which is a common phenomenon in the field of data science.

The class imbalance will cause “undo” effect because they cannot weaken the deviations of the majority class. Therefore, we used the SMOTE technique to alleviate the data imbalance problem, which synthesizes new minority instances from the nearest neighbors of a straight line connecting a small number of samples. These new instances are created based on the characteristics of the original dataset, so they become similar to the original minority class instances [39]. The algorithm flow is as follows [40].

1. For each minority sample x, calculate its distance from the other samples in the minority class and get k-nearest neighbors.
2. Randomly select a number of samples from the k-nearest neighbors.
3. For each randomly chosen nearest neighbor x’, a new sample is synthesized according to the formula: xnew = x + rand(0,1)*(x’-x).

Determine the sampling multiplier based on the sample imbalance ratio and repeat the above process. Finally, a balanced dataset can be obtained.

### Model construction

Statistical analyses were performed using SPSS 26.0. The enumeration data were expressed as count (percentage) and processed with a Chi-square test, whereas the measurement data were presented as the means ± standard deviation and analyzed by t-test. Factors with a *p*-value <0.05 in the univariate binary logistic regression analysis were used as candidate factors to construct predictive models. In this study, machine learning algorithms were selected as the modeling methods for DFU minor amputation prediction. The whole dataset was randomly divided into the training set and the verification set according to the proportion of approximately 7:3, in which the training set was used to build the prediction models, and the verification set was used to verify and evaluate the performance of the models. Five machine learning algorithms were adopted to build risk prediction models, including DT, RF, LR, SVM and XGBoost. Among them, RF and XGBoost are integrated machine learning classifiers, and the remainder are single classifiers. In the process of training, the optimal parameters were determined by 10 cross-validations.

### Model evaluation

The evaluation indicators, including accuracy, precision, recall rate, F1-score, and AUC, were calculated to assess the constructed models. The closer the values are to 1, the better the performance of the prediction model. In addition, our experiment also used the receiver operating characteristic curve (ROC) to graphically represent the discernibility. As the most persuasive measurement for predictive analysis in machine learning, we also employed the confusion matrix (CM) for further model evaluation, which is a summarized table of the number of actual values and the predicted values yielded by the prediction model. Details of all these evaluation metrics are shown in Table 1.

**Table 1:** Calculation formulae for the evaluation indicators of the model.

CM	Labeled	Predicted as negative	Predicted as positive
	Negative	TN	FP
	Positive	FN	TP
Recall	$\frac{TP}{TP+\;FN}$		
Accuracy	$\frac{TP+TN}{TP+FP+TN+FN}$		
Precision	$\frac{TP}{TP+FP}$		
F1-score	$\frac{2TP}{2TP+\;FP+FN}$		

***Abbreviations***: CM, confusion matrix; TP, true positive; FP, false positive; FN, false negative; TN, true negative.

## Results

### Patient characteristics

From January 2018 to December 2019, a total of 362 patients with Texas grade 3 DFU were collected, including 257 males and 105 females aged 26–88 years. All the patient data, including demographics and disease and treatment characteristics, grouped by amputation are listed in S2 Table.

### Univariable analysis results

Univariate regression analysis was conducted based on 362 patients with minor amputation as dependent variables, and demographics, wound characteristics, and laboratory indicators served as independent variables. Significant results are shown in Table 2, and 9 characteristic variables were selected (*P*<0.05): random blood glucose, years with diabetes, cardiovascular disease, peripheral arterial disease, smoking history, albumin, serum creatinine, C-reactive protein, and DFU history.

**Table 2:** Results of univariate analysis of minor amputation for diabetic foot ulcer patients.

	Non-amputation (n = 287)	Minor Amputation (n = 75)	Statistics(χ2)	*p*-values
Random blood glucose (mmol/L)			43.323	<0.001
<11.1	172(92.0%)	15(8.0%)		
11.1–16.7	69(60.5%)	45(39.5%)		
>16.7	46(75.4%)	15(24.6%)		
Years with diabetes			7.389	0.025
0–10	161(84.7%)	29(15.3%)		
11–20	99(73.9%)	35(26.1%)		
>20	27(71.1%)	11(28.9%)		
Cardiovascular diseases			4.453	0.035
No	71(87.7%)	10(12.3%)		
Yes	216(76.9%)	65(23.1%)		
Peripheral arterial diseases			8.949	0.003
No	78(90.7%)	8(9.3%)		
Yes	209(75.7%)	67(24.3%)		
Smoking history			7.600	0.006
No	131(86.2%)	21(13.8%)		
Yes	156(74.3%)	54(25.7%)		
Albumin(g/L)			12.361	0.002
35.0–50.0	121(87.1%)	18(12.9%)		
25.0–34.9	143(76.9%)	43(23.1%)		
<25.0	23(62.2%)	14(37.8%)		
Creatinine(μmol/L)			10.337	0.026
54–133	234(81.5%)	53(18.5%)		
134–186	22(71.0%)	9(29.0%)		
187–451	16(76.2%)	5(23.8%)		
452–771	11(84.6%)	2(15.4%)		
>771	4(40.0%)	6(60.0%)		
C-reactive protein(mg/L)			15.227	<0.001
0–8	124(89.9%)	14(10.1%)		
8–100	125(73.1%)	46(26.9%)		
>100	38(71.7%)	15(28.3%)		
DFU history			7.350	0.007
No	242(82.0%)	53(18.0%)		
Yes	45(67.2%)	22(32.8%)		

***Abbreviations***: DFU, diabetic foot ulcers.

### SMOTE algorithm

To solve the class imbalance of the binary variables, we adopted the SMOTE technique. We sampled both the training dataset and the verification dataset, and the sample distribution before and after oversampling is shown in Table 3.

**Table 3:** Data distribution of both the training set and validation set before and after SMOTE.

	Training set		Validation set	
	Non-amputation	Minor amputation	Non-amputation	Minor amputation
Before over-sampling	201	52	86	23
After over-sampling	201	201	86	86

***Abbreviations***: SMOTE, synthetic minority oversampling technique.

### Model establishment and evaluation

Undergoing a minor amputation operation or not was set as the label, and 9 statistically significant factors in univariate analysis were set as features. The risk prediction models of minor amputation in DFU patients were established by DT, RF, LR, SVM and XGBoost.

**Fig 2:**
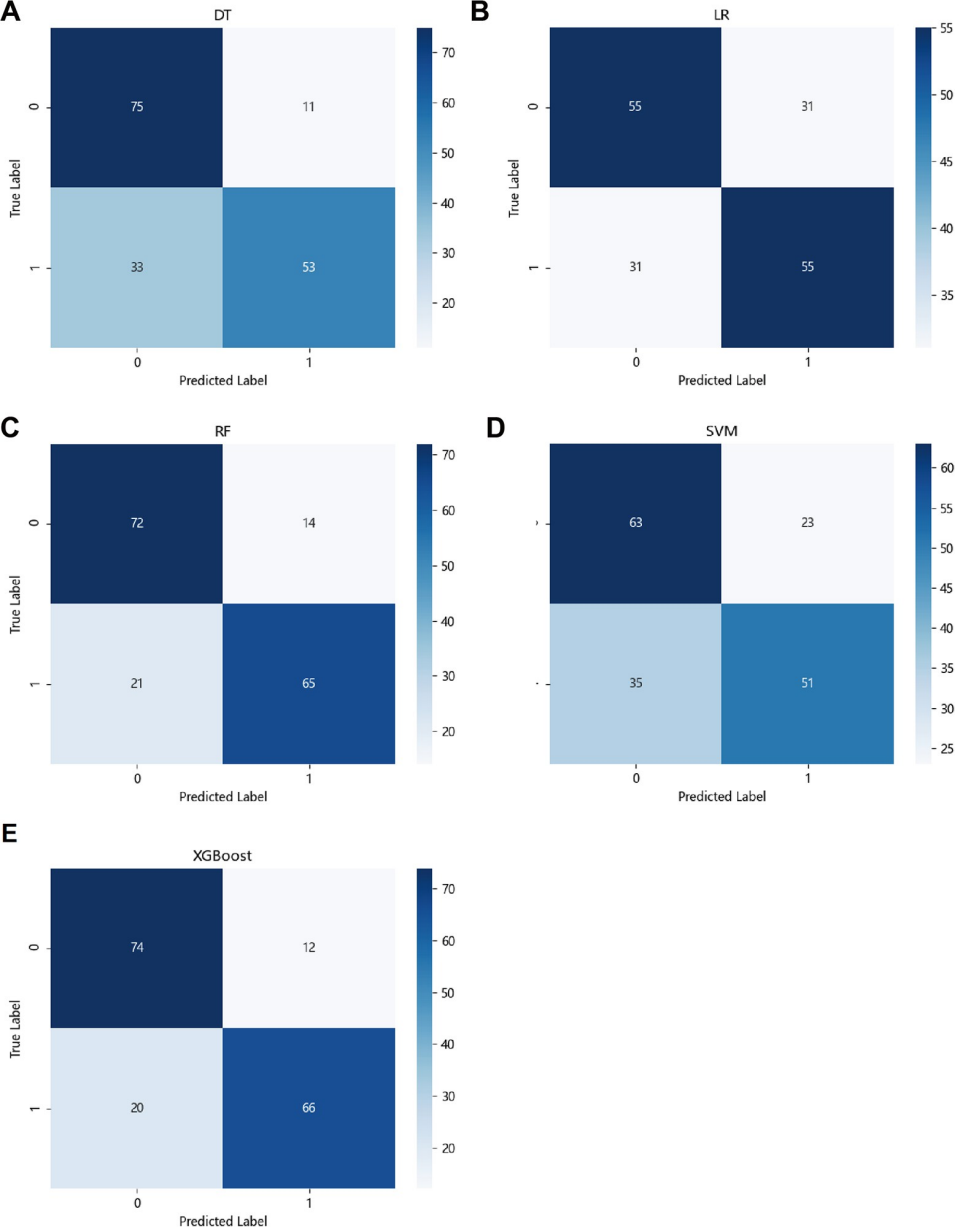
Confusion matrix of the risk prediction models with machine learning algorithms. (A) DT: decision tree. (B) RF: random forest. (C) LR: logistic regression. (D) SVM: support vector machine. (E) XGBoost: extreme gradient boosting.

Fig 2 shows the results of the confusion matrix of various models. The confusion matrix of the classification results consists of four quadrants. In our study, 0 represents patients who did not receive minor amputation during this treatment, and 1 represents patients who received minor amputation during this treatment.

**Fig 3:**
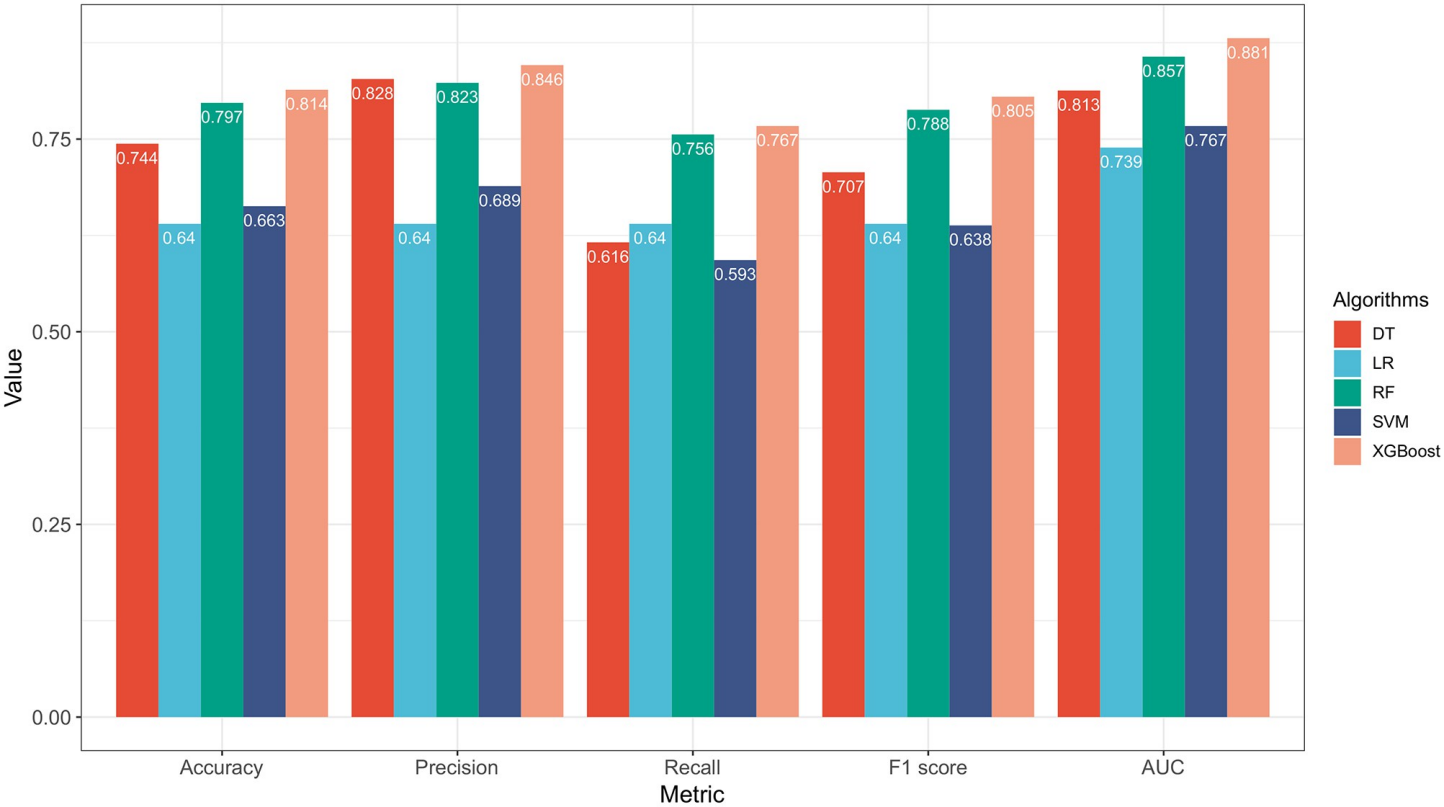
Performance comparisons of machine learning algorithms after over-sampling. ***Abbreviations***: DT, Decision Tree; RF, Random Forest; LR, Logistic Regression; SVM, Support Vector Machine; XGBoost, extreme gradient boosting; AUC, area under the curve.

**Fig 4:**
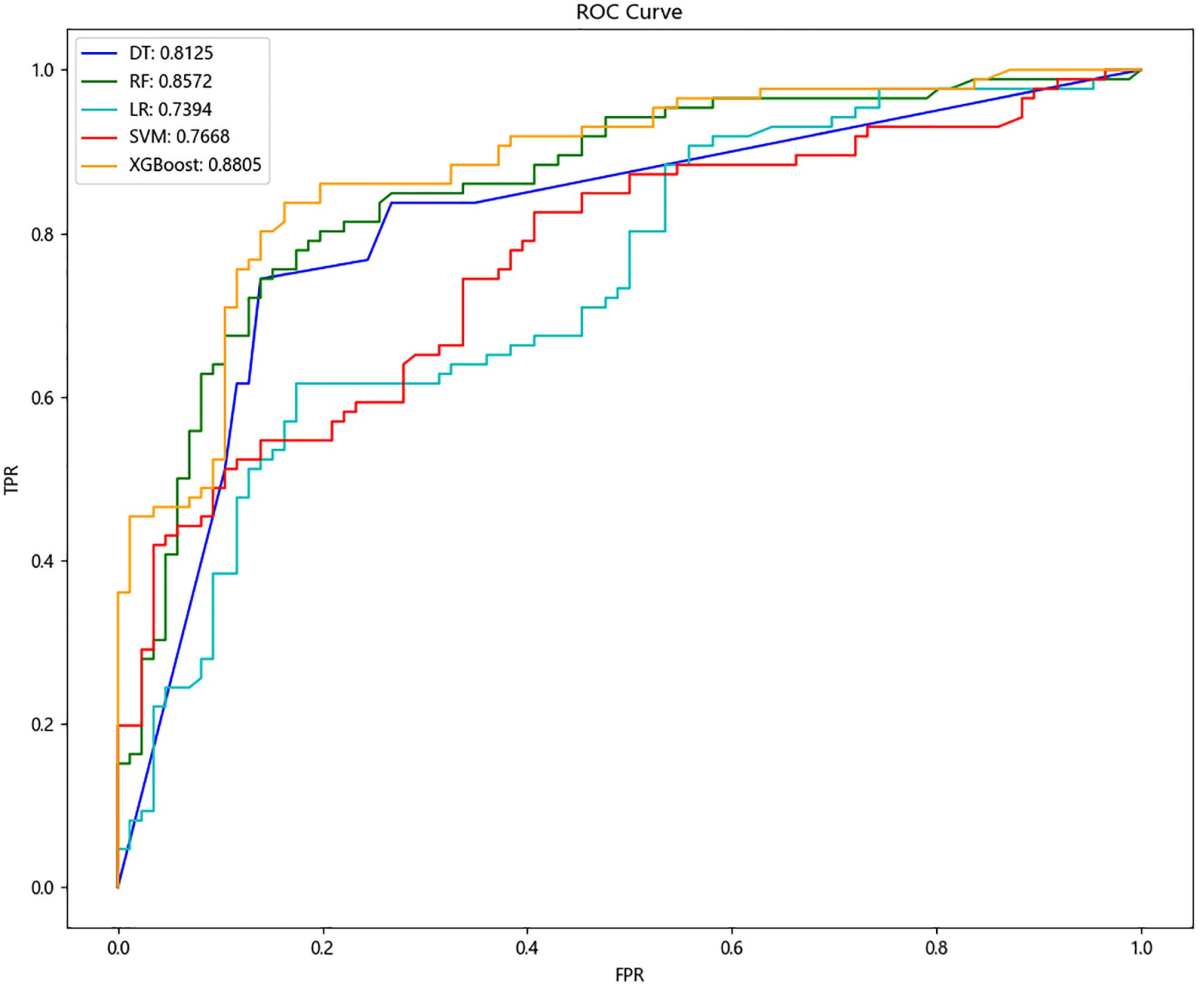
ROC curves for predicting minor amputation in DFU patients with machine learning algorithms after over-sampling. ***Abbreviations***: ROC, receiver operating characteristic curve; DFU, diabetic foot ulcers; DT, decision tree; RF, random forest; LR, logistic regression; SVM, support vector machine; XGBoost, extreme gradient boosting; FPR, false positive rate; TPR, true positive rate.

Firstly, we compared the prediction performance of five machine learning algorithms before and after oversampling as shown in Table 4, which demonstrates the effectiveness of the SMOTE algorithm. Before SMOTE technique, all five machine learning algorithms suffered from data imbalance, resulting in biased classification boundaries. Although the overall classification accuracy was satisfactory, other metrics, such as precision, recall, and F1 score, were significantly lower, proving that the obtained models were clinically meaningless in practice. To further clearly compare the performance of the five machine learning algorithms with SMOTE technique, we visualized the evaluation metrics shown in Figs 3 and 4. It is not difficult to find that the integrated algorithms XGBoost and RF were higher than other single classification algorithms from an overall perspective, and XGBoost had the highest value in all indicators (accuracy 0.814, precision 0.846, recall 0.767, F1-score 0.805, and AUC 0.881), which indicates that XGBoost obtained optimal performance in the prediction of minor amputation in UT3 diabetic foot ulcer.

**Table 4:** Performance parameter values for five machine learning algorithms before and after over-sampling.

	Algorithms	Accuracy	Precision	Recall	F1 score	AUC
Before oversampling	DT	0.743	0.333	0.217	0.263	0.688
	RF	0.780	0.455	0.217	0.294	0.754
	LR	0.771	0.429	0.261	0.324	0.733
	SVM	0.798	0.667	0.087	0.154	0.712
	XGBoost	0.817	0.615	0.348	0.444	0.726
After oversampling	DT	0.744	0.828	0.616	0.707	0.813
	RF	0.797	0.823	0.756	0.788	0.857
	LR	0.640	0.640	0.640	0.640	0.739
	SVM	0.663	0.689	0.593	0.638	0.767
	XGBoost	0.814	0.846	0.767	0.805	0.881

***Abbreviations***: AUC, area under the curve; DT, decision tree; RF, random forest; LR, logistic regression; SVM, support vector machine; XGBoost, extreme gradient boosting.

### Web-based calculator

Based on our dataset and the XGBoost algorithm, we constructed a web calculator using Microsoft Azure Web Sites. The address of the website is (https://dfuprediction.azurewebsites.net/). This web application enables the user to select nine variables to calculate the likelihood of minor amputation for DFU patients. S1 Fig contains an example.

### The importance of characteristic variables

**Fig 5:**
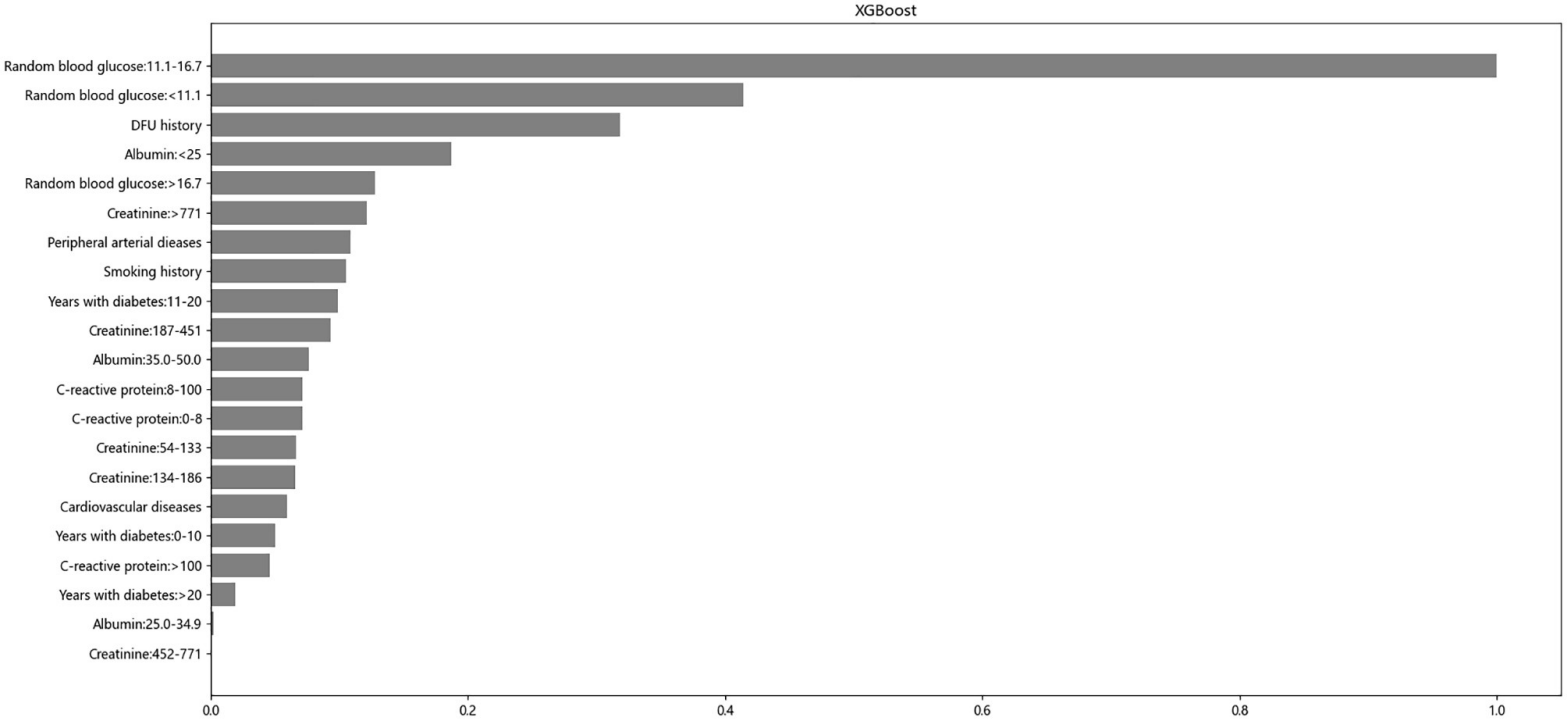
Feature importance ranking of the included feature of the XGBoost model. ***Abbreviations***: XGBoost, extreme gradient boosting.

Based on the algorithm evaluation, XGBoost had the strongest prediction ability. Thus, we explored the relative importance of each feature variable in the XGBoost risk model. The feature importance ranking results are shown in Fig 5. Random blood glucose occupied the first, second and fifth places of importance, and history of DFU and serum albumin values less than 25 g/L ranked third and fourth in importance, respectively. Other important features included creatinine >771 μmol/L, peripheral artery disease, smoking history, diabetes mellitus, CRP and cardiovascular disease.

## Discussion

In recent years, a variety of linear regression models have been established to obtain the prognosis of clinical diseases. Linear regression models mainly refer to logistic regression and Cox regression. These models have strong predictability for the outcome of classified variables, but the predictive ability may be reduced because these models ignore the nonlinear relationship. With the continuous development of artificial intelligence, machine learning has been applied in the field of medical diagnosis and demonstrated better performance in the prediction of clinical disease prognosis [41]. In recent studies, many studies have shown the superior predictive power of machine learning algorithms for the diagnosis and prognosis of DFU [14–19]. However, there is a lack of studies with sufficient case data to determine comparatively which machine learning algorithm is the most suitable for use, especially in patients with UT3 DFU, which is most commonly seen in tertiary care hospitals. Our study focuses on predicting the rate of minor amputations in patients with UT3 DFU to more efficiently achieve limb preservation and reduce complications.

To identify the most suitable model for predicting minor amputation in UT3 DFU patients, our team collected 21 factors based on demographic features, wound features, and laboratory indicators in 362 cases. Five algorithms, namely DT, RF, LR, SVM and XGBoost, were used to predict the minor amputation probability. The XGBoost algorithm performed better than the commonly used linear regression model (LR), the single classifier (DT and SVM), and another ensemble learning machine learning algorithm (RF). Thus, the XGBoost algorithm was best to predict minor amputation of DFU patients (Table 4, Figs 3, 4). XGBoost is an ensemble learning algorithm that combines the predictions of multiple trees and adds the predicted scores of each tree to obtain the final score. With the advantages of high speed, high efficiency and high fault tolerance, XGBoost can effectively avoid overfitting and exhibit a high generalization ability. Many researchers have demonstrated that XGBoost has achieved good performance in the prediction of a variety of clinical diseases [42, 43]. Our study also demonstrates the good operation of SMOTE oversampling in solving the balance class problem in small samples of medical data.

In the results of our feature importance analysis, the top five important factors in our ranking were random blood glucose, DFU history and albumin. Random blood glucose accounted for three of the top five factors, emphasizing the importance of blood glucose control. Some studies have shown that intensive blood glucose control may lead to hypoglycemia [44–47]. Nevertheless, the relaxation of blood glucose control has contributed to the return of LEA based on an analysis of adult participants in the National Health and Nutrition Survey (NHANES) from 2010 to 2015 [48]. The Action to Control Cardiovascular Risk in Diabetes (ACCORD) trial collected 10251 randomly selected type 2 diabetic patients at 77 locations in the United States and Canada. This trial found that intensive blood glucose therapy was associated with a 31% reduced risk of major lower limb amputation [49]. Based on a systematic review of the literature on diabetic foot disease, the guidelines of the Society of Vascular Surgery (SVS) recommend enhanced blood glucose control (HbA1c = 6–7.5%) to reduce the risk of amputation [50]. Paying early attention to strict blood glucose control may be a key factor in preventing the progression of severe limb ischemia and subsequent major limb amputation. Our research particularly emphasizes this point.

The remaining top five important factors in our ranking were DFU history and albumin. Byung-Joon Jeon et al. demonstrated that a history of previous DFU increased the risk of LEA (*P* = 0.008, OR = 3.38) [51]. In our study, 32.8% of patients with a history of diabetic ulcers underwent a minor amputation. Our results are similar to those of Helm et al., who reported that 20–58% of patients relapsed within one year of wound healing [52]. However, Jiang et al. observed in a cohort of 669 Chinese people that the amputation rate of diabetic DFU patients within two years was 19.03% [8]. Our data differed because as a cross-sectional study, the interval between the last occurrence of ulcers in our patients may be greater than 2 years, and all patients in our cohort were UT3 patients whose ulcers were severe enough to reach the tendon. Patients with a history of ulcers are likely to have all the risk factors for ulcers. This finding reminds us to follow up on DFU after treatment, including disease care education and regular reexamination.

Low serum albumin always indicates malnutrition, which is more common in patients with DFU given elevated flux and poorly controlled blood glucose. Adequate nutrition is essential for tissue remodeling. The GNRI score for assessing nutrition-related risks consists of serum albumin, body weight, and ideal weight. In a study on nutrition and DFU healing, foot prognosis deteriorated with the increase in the risk of malnutrition (GNRI score) (linear trend, *P*<0.001) and the incidence of major LEA (11.3% vs. 1.8%) in the malnutrition (GNRI<92) group was approximately 6-fold increased [53, 54]. Meanwhile, a serum albumin level of <25 g/L, indicating severe malnutrition, has a greater impact on the amputation rate of DFU. In this case, even if DFU is treated, the prognosis is still poor [38]. The results of a nutritional supplement study showed improvement in patients with DFU who received arginine, glutamine and β-hydroxy-β-methyl butyrate supplements with albumin <40 g/L rather than below the normal value of 55 g/L. More research is needed in this area because it is still not clear whether the infection worsened due to the patient’s long-term hypoproteinemia or whether the serum albumin decreased sharply when dealing with the infection [55].

Our research is characterized by the following points: 1. Our study provides a real-world dataset from two tertiary hospitals in East China, focusing on outcomes in patients with UT3 diabetic foot ulcers and minor amputations. 2. We compared five popular machine learning algorithms (LR, RF, DT, SVM and XGBoost) to filter out the best-performing clinical models and ultimately provided a network calculator for use (https://dfuprediction.azurewebsites.net/). 3. Our study showed good results in small sample medical data using an oversampling approach (SMOTE) to balance the data. 4. Based on the results of the ranking of factors affecting the risk of minor amputation, we suggest that strict glycaemic control, active DFU follow-up including disease care education and regular review, and improved malnutrition are three key points to avoid minor amputation.

This study has some limitations. First, this study collected case data from the *** Hospital and the *** Hospital. Because these hospitals serve as the main referral centers for DFU, our conclusions are sufficiently representative. However, the amount of data is limited, limiting the performance of machine learning. We aim to verify the results in a larger sample size of patients from multiple centers in the future. Finally, due to incomplete examination records, we did not include some laboratory indicators that may be related to minor amputation, such as glycosylated hemoglobin and serum insulin levels, or the treatment of other diseases of the patient, such as hypolipidemic agents for ischemic heart disease and anticoagulants for peripheral vascular disease. The field of artificial intelligence is developing rapidly. Data from multisensory data and imaging CT and MRI data are already being used extensively in medical decision-making [56–58]. In the field of diabetic foot ulcers, we look forward to incorporating such data in our follow-up work to make more accurate monitoring and timely treatment of patients.

## Conclusion

In this study, we used the data of patients with UT3 DFU collected from two tertiary care hospitals to train, test, and evaluate the predictive ability of five machine learning algorithms for minor amputation. Ultimately, XGBoost shows the best performance. The web calculator we have created would help clinical workers assess the risk of small amputation of diabetic foot ulcers at the time of admission, implement individualized treatment and optimize patient outcomes.

## Supporting information

S1 Fig An example of using the web calculator. (TIF)

S1 Table University of Texas classification system. (DOCX)

S2 Table Differences between demographic and clinical characteristics of non-amputation and minor amputation groups. (DOCX)

S1 Checklist STROBE statement—Checklist of items that should be included in reports of observational studies. (DOCX)
