# Supplementary figures and images for: Machine learning for the prediction of minor amputation in University of Texas grade 3 diabetic foot ulcers
Source: PLoS One. 2022 Dec 6;17(12):e0278445. doi: 10.1371/journal.pone.0278445 (PMC9725167; doi:10.1371/journal.pone.0278445)

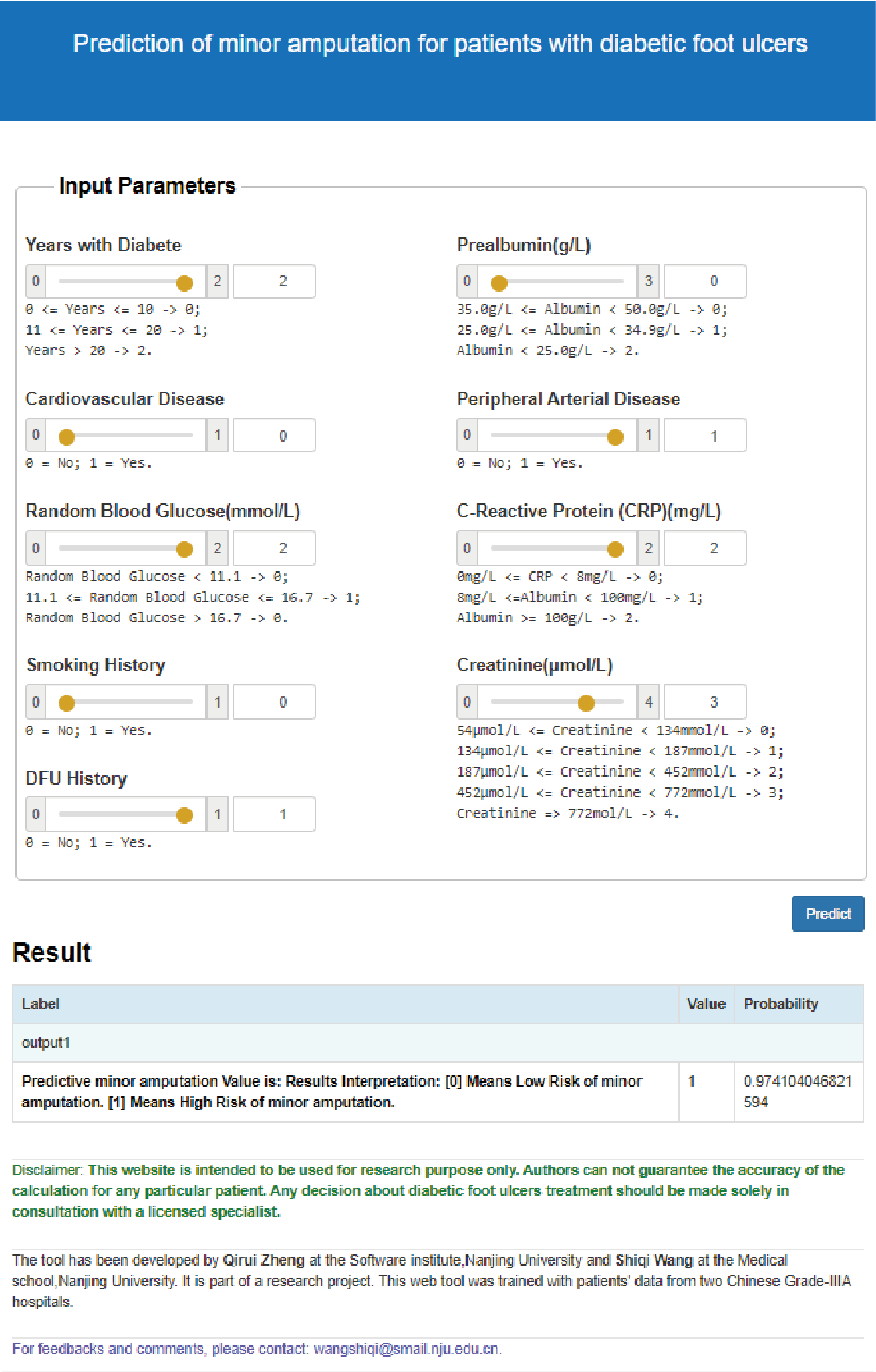

Supplement: S1 Fig — (TIF) [file pone.0278445.s001.tif]
