# Supplementary material for: Machine learning for the prediction of minor amputation in University of Texas grade 3 diabetic foot ulcers
Source: PLoS One. 2022 Dec 6;17(12):e0278445. doi: 10.1371/journal.pone.0278445 (PMC9725167; doi:10.1371/journal.pone.0278445)
Supplement: S1 Table — (DOCX) [file pone.0278445.s002.docx]

| Stage | Grade | | | |
| --- | --- | --- | --- | --- |
|  | 0 | 1 | 2 | 3 |
| A | Preulcerative or postulcerative  lesion completely epithelized | Superficial wound, not involving  tendon, capsule, or bone | Wound penetrating to  tendon or capsule | Wound penetrating to bone or joint |
| B | Infected | Infected | Infected | Infected |
| C | Ischemic | Ischemic | Ischemic | Ischemic |
| D | Infected and Ischemic | Infected and Ischemic | Infected and Ischemic | Infected and Ischemic |
