# Supplementary material for: Machine learning for the prediction of minor amputation in University of Texas grade 3 diabetic foot ulcers
Source: PLoS One. 2022 Dec 6;17(12):e0278445. doi: 10.1371/journal.pone.0278445 (PMC9725167; doi:10.1371/journal.pone.0278445)
Supplement: S2 Table — (DOCX) [file pone.0278445.s003.docx]

|  | Non-amputation (n=287) | Minor amputation (n=75) | Statistics(χ2) | *p*-values |
| --- | --- | --- | --- | --- |
| Age (years) |  |  | 5.311 | 0.257 |
| ≤49 | 29(82.9%) | 6(17.1%) |  |  |
| 50-59 | 73(76.0%) | 23(24.0% |  |  |
| 60-69 | 79(74.5%) | 27(25.5%) |  |  |
| 70-79 | 79(83.2%) | 16(16.8% |  |  |
| 80-89 | 27(90.0%) | 3(10.0%) |  |  |
| Sex |  |  | 0.860 | 0.354 |
| Female | 80(76.2%) | 25(23.8%) |  |  |
| Male | 207(80.5%) | 50(19.5%) |  |  |
| Current use of Insulin |  |  | 1.142 | 0.285 |
| No | 91(82.7%) | 19(17.3%) |  |  |
| Yes | 196(77.8%) | 56(22.2%) |  |  |
| Random blood glucose(mmol/L) |  |  | 43.323 | <0.001 |
| <11.1 | 172(92.0%) | 15(8.0%) |  |  |
| 11.1-16.7 | 69(60.5%) | 45(39.5%) |  |  |
| >16.7 | 46(75.4%) | 15(24.6%) |  |  |
| Years of diabetes |  |  | 7.389 | 0.025 |
| 0-10 | 161(84.7%) | 29(15.3%) |  |  |
| 11-20 | 99(73.9%) | 35(26.1%) |  |  |
| >20 | 27(71.1%) | 11(28.9%) |  |  |
| Wound area(cm^2^) |  |  | 5.450 | 0.134 |
| 0-4 | 146(80.7%) | 35(19.3%) |  |  |
| 5-16 | 103(75.7%) | 33(24.3%) |  |  |
| 17-25 | 17(73.9%) | 6(26.1%) |  |  |
| >25 | 21(95.5%) | 1(4.5%) |  |  |
| Wound duration |  |  | 2.547 | 0.635 |
| <1w | 57(81.4%) | 13(18.6%) |  |  |
| 1w-1m | 133(76.4%) | 41(23.6%) |  |  |
| 1m-3m | 48(81.4%) | 11(18.6%) |  |  |
| 3m-6m | 33(86.8%) | 5(13.2%) |  |  |
| >6m | 16(76.2%) | 5(23.8%) |  |  |
| Wound classification(UT) |  |  | 4.633 | 0.179 |
| A3 | 6(100.0%) | 0(0.0%) |  |  |
| B3 | 135(80.8%) | 32(19.2%) |  |  |
| C3 | 21(91.3%) | 2(8.7%) |  |  |
| D3 | 125(75.3%) | 41(24.7%) |  |  |
| Ulcer location |  |  | 8.765 | 0.109 |
| Toes and interphalangeal | 139(75.1%) | 46(24.9%) |  |  |
| Dorsal foot | 40(80.0%) | 10(20.0%) |  |  |
| Pedal foot | 40(78.4%) | 11(21.6%) |  |  |
| Heel | 12(100.0%) | 0(0.0%) |  |  |
| Full foot | 36(83.7%) | 7(16.3%) |  |  |
| Ankle and Shin | 20(95.2%) | 1(4.8%) |  |  |
| Hyperlipidemia |  |  | 1.255 | 0.263 |
| No | 106(76.3%) | 33(23.7%) |  |  |
| Yes | 181(81.2%) | 42(18.8%) |  |  |
| Retinopathy |  |  | 0.574 | 0.448 |
| No | 230(78.5%) | 63(21.5%) |  |  |
| Yes | 57(82.6%) | 12(17.4%) |  |  |
| Cardiovascular diseases |  |  | 4.453 | 0.035 |
| No | 71(87.7%) | 10(12.3%) |  |  |
| Yes | 216(76.9%) | 65(23.1%) |  |  |
| Peripheral arterial diseases |  |  | 8.949 | 0.003 |
| No | 78(90.7%) | 8(9.3%) |  |  |
| Yes | 209(75.7%) | 67(24.3%) |  |  |
| Neurologic disease |  |  | 0.104 | 0.747 |
| No | 174(78.7%) | 47(21.3%) |  |  |
| Yes | 113(80.1%) | 28(19.9%) |  |  |
| Hyperlipidemia |  |  | 2.247 | 0.134 |
| No | 209(81.3%) | 48(18.7%) |  |  |
| Yes | 78(74.3%) | 27(25.7%) |  |  |
| Drinking History |  |  | 1.840 | 0.175 |
| No | 238(78.0%) | 67(22.0%) |  |  |
| Yes | 49(86.0%) | 8(14.0%) |  |  |
| Smoking History |  |  | 7.600 | 0.006 |
| No | 131(86.2%) | 21(13.8%) |  |  |
| Yes | 156(74.3%) | 54(25.7%) |  |  |
| Albumin(g/L) |  |  | 12.361 | 0.002 |
| 35.0-50.0 | 121(87.1%) | 18(12.9%) |  |  |
| 25.0-34.9 | 143(76.9%) | 43(23.1%) |  |  |
| <25 | 23(62.2%) | 14(37.8%) |  |  |
| Creatinine(μmol/L) |  |  | 10.337 | 0.026 |
| 54-133 | 234(81.5%) | 53(18.5%) |  |  |
| 134-186 | 22(71.0%) | 9(29.0%) |  |  |
| 187-451 | 16(76.2%) | 5(23.8%) |  |  |
| 452-771 | 11(84.6%) | 2(15.4%) |  |  |
| >771 | 4(40.0%) | 6(60.0%) |  |  |
| C-reactive protein (mg/L) |  |  | 15.227 | <0.001 |
| 0-8 | 124(89.9%) | 14(10.1%) |  |  |
| 8-100 | 125(73.1%) | 46(26.9%) |  |  |
| >100 | 38(71.7%) | 15(28.3%) |  |  |
| History |  |  | 7.350 | 0.007 |
| No | 242(82.0%) | 53(18.0%) |  |  |
| Yes | 45(67.2%) | 22(32.8%) |  |  |
