# Supplementary material for: Machine learning for the prediction of minor amputation in University of Texas grade 3 diabetic foot ulcers
Source: PLoS One. 2022 Dec 6;17(12):e0278445. doi: 10.1371/journal.pone.0278445 (PMC9725167; doi:10.1371/journal.pone.0278445)
Supplement: S1 Checklist — (DOCX) [file pone.0278445.s004.docx]

STROBE Statement—checklist of items that should be included in reports of observational studies

|  | Item No. | Recommendation | Page  No. | Relevant text from manuscript |
| --- | --- | --- | --- | --- |
| **Title and abstract** | 1 | (*a*) Indicate the study’s design with a commonly used term in the title or the abstract | 2 | We aim to establish a predictive model based on machine learning to quickly identify patients requiring minor amputation among newly admitted patients with DFU. |
|  |  | (*b*) Provide in the abstract an informative and balanced summary of what was done and what was found | 2 | Overall, 362 cases with University of Texas grade 3 DFU were screened from tertiary care hospitals in East China. We employed the synthetic minority oversampling technique for the unevenness of the original dataset. Then, five risk prediction models based on decision tree, random forest, logistic regression, support vector machine, and extreme gradient boosting (XGBoost) were separately constructed by Python. Univariable analysis identified 9 factors for inclusion in the model: random blood glucose, years with diabetes, cardiovascular diseases, peripheral arterial diseases, DFU history, smoking history, albumin, creatinine, and C-reactive protein. After model evaluation, XGBoost obtained the best performance with the highest score (accuracy 0.814, precision 0.846, recall 0.767, F1-score 0.805, and AUC 0.881). These results suggested XGBoost can be used to construct an effective prediction model for minor amputation in Texas grade 3 DFU patients, and may help clinicians assess the risk of minor amputation at first admission. |
| Introduction | | | |  |
| Background/rationale | 2 | Explain the scientific background and rationale for the investigation being reported | 3-5 | It is estimated that the number of diabetes patients worldwide will reach 300 million by 2025[1]. Diabetic ulcers, especially severe diabetic foot ulcers (DFU), are the most common serious complication of diabetes. Debridement and repair are common treatments for DFU patients, and amputation may be necessary if the wound level is severe, which has been an important cause of nontraumatic amputation. Approximately 50-70% of patients who need amputation due to nontraumatic injury are diabetic patients[2]. Clinical practitioners often evaluate DFUs using several wound grading systems, of which the University of Texas grading is a widely used evaluation system featuring detection of infection, ischemia and depth of penetration into tissue, details of which can be seen in the S1 Table. The risk of amputation is highly correlated with the grade of the wound, and patients with a University of Texas grade 3 (penetration to bone or joint) DFU are 11 times more likely to face amputation[3]. Amputation can have a serious impact on the prognosis of patients with DFU, which not only reduces the quality of life but also increases the risk of death[4-6]. According to statistics, the 5-year mortality rate for DFU patients with amputation is as high as 63%, compared with 40% for patients with DFU[1]. Moreover, amputation further increases the cost of treatment by fivefold which adds more burden to national health care[7, 8]. DFU amputation can be classified into minor amputation below the ankle joint and major amputation above the ankle level. In contrast to major amputation as a life-saving procedure, minor amputation is a choice for local limbs that are difficult to save and accounts for a larger clinical proportion of patients who have amputation performed (approximately 90%)[9]. Early identification of DFU minor amputation is very important for DFU patients, especially those with severe wounds (Texas grade 3), to avoid major amputation and save limbs[10, 11].  Machine learning is a subfield of artificial intelligence that has brought revolutionary changes to the field of health care through fast, efficient, accurate, and cost-effective computing decisions[12]. Machine learning plays an important role in the prediction of many common diseases, such as the diagnostic prediction of patients with type 2 diabetes[13] and the classification of cardiovascular diseases in patients with diabetes[14]. Most machine learning algorithms can be regarded as mathematical models that map a set of observation variables (i.e., features or predictors) to a set of result variables (i.e., tags or targets)[15] According to the number and type of supervision received during training, machine learning can be divided into the following four types: supervised learning, unsupervised learning, semisupervised learning, and reinforcement learning. Classification (discrete class label) and regression (continuous value label) are two categories in supervised learning. At present, the main supervised learning algorithms used in the biomedical field include linear regression, logistic regression (LR), naïve Bayesian, support vector machine (SVM), random forest (RF), and decision tree (DT)[12]. In a particular scenario, the choice of a machine learning algorithm depends on the sample dataset and the decision objective. Several studies on DFU have shown the superior predictive performance of machine learning algorithms [16-21]. For the DFU diagnosis, Amith Khandakar et al. used thermogram images to establish a machine learning model based on CNN for early detection of DFU[22]. Rachita Nanda et al. constructed the prediction models for differentiating T2DM with DFU with four distinct machine learning methods[19]. For the DFU prognosis prediction, the Bayesian-based decision model[23] and the light gradient boosting machine model[21] were used to predict amputation rate of DFU patients in different retrospective studies. Recently, a Chinese team focused on DFU mortality and amputation during the COVID-19 post lockdown compared different machine learning models[20]. However, these studies are limited by a lack of data, and the majority employ only a single machine learning technique. In terms of amputation prognosis in DFU, there is a dearth of studies comparing different machine learning techniques. |
| Objectives | 3 | State specific objectives, including any prespecified hypotheses | 5 | In this study, to help clinicians quickly and accurately identify patients with a Texas grade 3 DFU who require minor amputation, we used the following five supervised learning algorithms to filter the best prediction models: LR, a long-standing statistical method to predict patients’ results based on the predictive variables of each patient[24]; RF, a mature learning algorithm widely used based on recursive methods[25]; DT, a tree model in which each node has a question and each branch represents a result[26]; SVM, an excellent technology with independent integrity theory based on the optimal solution[27]; and extreme gradient boosting (XGBoost), an enhanced learning algorithm, which aims to transform weak learners into strong learners with high prediction accuracy[28]. |
| Methods | | | |  |
| Study design | 4 | Present key elements of study design early in the paper | 5 |  |
| Setting | 5 | Describe the setting, locations, and relevant dates, including periods of recruitment, exposure, follow-up, and data collection | 6 | All clinical data were selected from *** Hospital and the *** Hospital from January 2018 to December 2019. Both hospitals treated DFU under multidisciplinary cooperation. Only patients with Texas grade 3 DFU were included in the study while patients who received major amputation, gave up treatment, or had incomplete information were excluded. The demographic data, wound characteristics and laboratory indicators of patients were collected from the medical records. According to the Helsinki Declaration, our study was approved by the Ethics Committee of Nanjing Drum Tower Hospital (No. 2020-10901). Informed consent of the participants was waived because of the retrospective study design and the use of anonymized clinical data. |
| Participants | 6 | (*a*) *Cohort study*—Give the eligibility criteria, and the sources and methods of selection of participants. Describe methods of follow-up  *Case-control study*—Give the eligibility criteria, and the sources and methods of case ascertainment and control selection. Give the rationale for the choice of cases and controls  *Cross-sectional study*—Give the eligibility criteria, and the sources and methods of selection of participants | 6 | Only patients with Texas grade 3 DFU were included in the study while patients who received major amputation, gave up treatment, or had incomplete information were excluded. |
|  |  | (*b*) *Cohort study*—For matched studies, give matching criteria and number of exposed and unexposed  *Case-control study*—For matched studies, give matching criteria and the number of controls per case |  |  |
| Variables | 7 | Clearly define all outcomes, exposures, predictors, potential confounders, and effect modifiers. Give diagnostic criteria, if applicable | 6-8 | Outcome  A minor amputation is defined as any amputation distal to the ankle joint.  Predictors  A total of 21 variables that have been shown to have a prognostic impact on diabetic ulcers in the previous literature were collected[29, 30]. All the enrolled predictors can be seen in the flowchart (Fig 1). To avoid confusion, we have given specific explanations of the clinical indicators that may be controversial as follows:  Ulcer location: The location of the ulcer was judged by the endocrinologist and the burn plastic surgeon at the first visit to the hospital.  Wound duration: This period ranged from the first discovery to the first visit to the hospital.  Diabetic peripheral neuropathy (DPN): Clinical symptoms, such as finger/toe symmetrical sensory disturbance or abnormal nerve conduction velocity, are noted on electrophysiological nerve examination. Two of the following diagnostic criteria of DPN must be met[31]: (1) neuropathic pain, anesthesia, or other sensory abnormalities; (2) abnormal acupuncture sensation of the lower extremities or changes in the 10 g Sims-Weinstein monofilament test; or (3) decreased ankle reflex.  Smoking history: The definition refers to the standard recommended by the WHO in 1984, that is, smoking more than one cigarette a day for a continuous period of one month; otherwise, it is judged to be nonsmoking.  Drinking history: The criteria for being classified as a drinker includes a long history of drinking lasting for more than 5 years and daily alcohol consumption ≥ 80 g.  Hypertension: The criteria for determining hypertension are as follows: systolic blood pressure≥ 140/90 mmHg or use of antihypertensive drugs with normal blood pressure.  Peripheral artery disease (PAD): If one or more lower extremity artery occlusions are found by Doppler ultrasound, it is diagnosed as peripheral artery disease[32].  Hyperlipidemia: Hyperlipidemia is known as lipid metabolism disorder and refers to total cholesterol, triglyceride, high-density lipoprotein cholesterol and low-density lipoprotein cholesterol exceeding the standard value.  Fig 1. Feature selection, training and validation processes for the prediction models with machine learning algorithms.  To explore whether the variables were dose-dependent on the outcome of amputation of diabetic foot ulcers, we defined the abnormal values of variables according to clinical experience and guidelines[33-37]. Patients were divided based on 11.1-16.7 mmol/L and >16.7 mmol/L blood glucose. Patients were divided based on 25.0-34.9 g/L and < 25.0 g serum albumin levels. Patients were divided based on 8-100 mg/L and > 100 mg/L CRP levels. The duration of diabetes was divided into 0-10 years, 10-20 years and > 20 years. Patients were divided based on creatine levels as follows: 134-186 μmol/L, 187-451 μmol/L, 452-771 μmol/L and > 771 μmol/L. Details are provided in S2 Table. |
| Data sources/ measurement | 8* | For each variable of interest, give sources of data and details of methods of assessment (measurement). Describe comparability of assessment methods if there is more than one group | 6-8 | Outcome  A minor amputation is defined as any amputation distal to the ankle joint.  Predictors  A total of 21 variables that have been shown to have a prognostic impact on diabetic ulcers in the previous literature were collected[29, 30]. All the enrolled predictors can be seen in the flowchart (Fig 1). To avoid confusion, we have given specific explanations of the clinical indicators that may be controversial as follows:  Ulcer location: The location of the ulcer was judged by the endocrinologist and the burn plastic surgeon at the first visit to the hospital.  Wound duration: This period ranged from the first discovery to the first visit to the hospital.  Diabetic peripheral neuropathy (DPN): Clinical symptoms, such as finger/toe symmetrical sensory disturbance or abnormal nerve conduction velocity, are noted on electrophysiological nerve examination. Two of the following diagnostic criteria of DPN must be met[31]: (1) neuropathic pain, anesthesia, or other sensory abnormalities; (2) abnormal acupuncture sensation of the lower extremities or changes in the 10 g Sims-Weinstein monofilament test; or (3) decreased ankle reflex.  Smoking history: The definition refers to the standard recommended by the WHO in 1984, that is, smoking more than one cigarette a day for a continuous period of one month; otherwise, it is judged to be nonsmoking.  Drinking history: The criteria for being classified as a drinker includes a long history of drinking lasting for more than 5 years and daily alcohol consumption ≥ 80 g.  Hypertension: The criteria for determining hypertension are as follows: systolic blood pressure≥ 140/90 mmHg or use of antihypertensive drugs with normal blood pressure.  Peripheral artery disease (PAD): If one or more lower extremity artery occlusions are found by Doppler ultrasound, it is diagnosed as peripheral artery disease[32].  Hyperlipidemia: Hyperlipidemia is known as lipid metabolism disorder and refers to total cholesterol, triglyceride, high-density lipoprotein cholesterol and low-density lipoprotein cholesterol exceeding the standard value.  Fig 1. Feature selection, training and validation processes for the prediction models with machine learning algorithms.  To explore whether the variables were dose-dependent on the outcome of amputation of diabetic foot ulcers, we defined the abnormal values of variables according to clinical experience and guidelines[33-37]. Patients were divided based on 11.1-16.7 mmol/L and >16.7 mmol/L blood glucose. Patients were divided based on 25.0-34.9 g/L and < 25.0 g serum albumin levels. Patients were divided based on 8-100 mg/L and > 100 mg/L CRP levels. The duration of diabetes was divided into 0-10 years, 10-20 years and > 20 years. Patients were divided based on creatine levels as follows: 134-186 μmol/L, 187-451 μmol/L, 452-771 μmol/L and > 771 μmol/L. Details are provided in S2 Table. |
| Bias | 9 | Describe any efforts to address potential sources of bias | 8, 9 | SMOTE for the imbalanced dataset  Our dataset consisted of 75 (20.7%) patients with minor amputation and 287 (79.3%) patients without any amputation. Obviously, a class imbalance exists in the original dataset, which is a common phenomenon in the field of data science. However, the class imbalance will cause overfitted prediction results because they do not weaken the deviations of the majority class. Therefore, the “sampling method” is proposed to equal the distribution of categories of the dataset by increasing the number of minority class and/or decreasing the number of majority class[38]. In this study, we used the synthetic minority oversampling technique (SMOTE) to randomly generate new examples or instances of the minority class from the nearest neighbors of a straight line connecting a small number of samples to balance the dataset. These new instances are created based on the characteristics of the original dataset, so they become similar to the original minority class instances[39]. |
| Study size | 10 | Explain how the study size was arrived at | 6 | A total of 21 variables that have been shown to have a prognostic impact on diabetic ulcers in the previous literature were collected |

Continued on next page

| Quantitative variables | 11 | Explain how quantitative variables were handled in the analyses. If applicable, describe which groupings were chosen and why | 8 | To explore whether the variables were dose-dependent on the outcome of amputation of diabetic foot ulcers, we defined the abnormal values of variables according to clinical experience and guidelines[33-37]. Patients were divided based on 11.1-16.7 mmol/L and >16.7 mmol/L blood glucose. Patients were divided based on 25.0-34.9 g/L and < 25.0 g serum albumin levels. Patients were divided based on 8-100 mg/L and > 100 mg/L CRP levels. The duration of diabetes was divided into 0-10 years, 10-20 years and > 20 years. Patients were divided based on creatine levels as follows: 134-186 μmol/L, 187-451 μmol/L, 452-771 μmol/L and > 771 μmol/L. Details are provided in S2 Table. |
| --- | --- | --- | --- | --- |
| Statistical methods | 12 | (*a*) Describe all statistical methods, including those used to control for confounding | 8-11 | SMOTE for the imbalanced dataset  Our dataset consisted of 75 (20.7%) patients with minor amputation and 287 (79.3%) patients without any amputation. Obviously, a class imbalance exists in the original dataset, which is a common phenomenon in the field of data science. However, the class imbalance will cause overfitted prediction results because they do not weaken the deviations of the majority class. Therefore, the “sampling method” is proposed to equal the distribution of categories of the dataset by increasing the number of minority class and/or decreasing the number of majority class[38]. In this study, we used the synthetic minority oversampling technique (SMOTE) to randomly generate new examples or instances of the minority class from the nearest neighbors of a straight line connecting a small number of samples to balance the dataset. These new instances are created based on the characteristics of the original dataset, so they become similar to the original minority class instances[39].  Model construction  Statistical analyses were performed using SPSS 26.0. The enumeration data were expressed as count (percentage) and processed with a Chi-square test, whereas the measurement data were presented as the means ± standard deviation and analyzed by t-test. Factors with a p-value <0.05 in the univariate binary logistic regression analysis were used as candidate factors to construct predictive models. In this study, machine learning algorithms were selected as the modeling methods for DFU minor amputation prediction. The whole dataset was randomly divided into the training set and the verification set according to the proportion of approximately 7:3, in which the training set was used to build the prediction models, and the verification set was used to verify and evaluate the performance of the models. Five machine learning algorithms were adopted to build risk prediction models, including DT, RF, LR, SVM and XGBoost. Among them, RF and XGBoost are integrated machine learning classifiers, and the remainder are single classifiers. In the process of training, the optimal parameters were determined by 10 cross-validations. The process of filtering variables and building and evaluating models is shown in Fig 1.  Model evaluation  The evaluation indicators, including accuracy, precision, recall rate, F1-score, and AUC, were calculated to assess the constructed models. The closer the values are to 1, the better the performance of the prediction model. In addition, our experiment also used the receiver operating characteristic curve (ROC) to graphically represent the discernibility. As the most persuasive measurement for predictive analysis in machine learning, we also employed the confusion matrix (CM) for further model evaluation, which is a summarized table of the number of actual values and the predicted values yielded by the prediction model. Details of all these evaluation metrics are shown in Table 1. |
|  |  | (*b*) Describe any methods used to examine subgroups and interactions | 8 | Details are provided in S2 Table. |
|  |  | (*c*) Explain how missing data were addressed | 6 | Only patients with Texas grade 3 DFU were included in the study while patients who received major amputation, gave up treatment, or had incomplete information were excluded. |
|  |  | (*d*) *Cohort study*—If applicable, explain how loss to follow-up was addressed  *Case-control study*—If applicable, explain how matching of cases and controls was addressed  *Cross-sectional study*—If applicable, describe analytical methods taking account of sampling strategy | 11-13 | All the patient data, including demographics and disease and treatment characteristics, grouped by amputation are listed in Table 2. |
|  |  | (*e*) Describe any sensitivity analyses | 13 | Univariable analysis results  Univariate regression analysis was conducted based on 362 patients with minor amputation as dependent variables, and demographics, wound characteristics, and laboratory indicators served as independent variables. Significant results are shown in Table 2, and 9 characteristic variables were selected (P < 0.05): random blood glucose, years with diabetes, cardiovascular disease, peripheral arterial disease, smoking history, albumin, serum creatinine, C-reactive protein, and DFU history. Complete univariable analysis results of all characteristic variables are provided in S2 Table. |
| Results | | | | |
| Participants | 13* | (a) Report numbers of individuals at each stage of study—eg numbers potentially eligible, examined for eligibility, confirmed eligible, included in the study, completing follow-up, and analysed | 11 | From January 2018 to December 2019, a total of 362 patients with Texas grade 3 DFU were collected, including 257 males and 105 females aged 26-88 years. All the patient data, including demographics and disease and treatment characteristics, grouped by amputation are listed in Table 2. |
|  |  | (b) Give reasons for non-participation at each stage |  |  |
|  |  | (c) Consider use of a flow diagram | 8 | Fig 1. Feature selection, training and validation processes for the prediction models with machine learning algorithms. |
| Descriptive data | 14* | (a) Give characteristics of study participants (eg demographic, clinical, social) and information on exposures and potential confounders | 8,11 | Fig 1. Feature selection, training and validation processes for the prediction models with machine learning algorithms.  From January 2018 to December 2019, a total of 362 patients with Texas grade 3 DFU were collected, including 257 males and 105 females aged 26-88 years. All the patient data, including demographics and disease and treatment characteristics, grouped by amputation are listed in Table 2. |
|  |  | (b) Indicate number of participants with missing data for each variable of interest | 6 | Only patients with Texas grade 3 DFU were included in the study while patients who received major amputation, gave up treatment, or had incomplete information were excluded. |
|  |  | (c) *Cohort study*—Summarise follow-up time (eg, average and total amount) | Not applicable |  |
| Outcome data | 15* | *Cohort study*—Report numbers of outcome events or summary measures over time |  |  |
|  |  | *Case-control study—*Report numbers in each exposure category, or summary measures of exposure |  |  |
|  |  | *Cross-sectional study—*Report numbers of outcome events or summary measures | 11 | From January 2018 to December 2019, a total of 362 patients with Texas grade 3 DFU were collected, including 257 males and 105 females aged 26-88 years. |
| Main results | 16 | (*a*) Give unadjusted estimates and, if applicable, confounder-adjusted estimates and their precision (eg, 95% confidence interval). Make clear which confounders were adjusted for and why they were included | 11 | Univariate regression analysis was conducted based on 362 patients with minor amputation as dependent variables, and demographics, wound characteristics, and laboratory indicators served as independent variables. Significant results are shown in Table 2, and 9 characteristic variables were selected (P < 0.05): random blood glucose, years with diabetes, cardiovascular disease, peripheral arterial disease, smoking history, albumin, serum creatinine, C-reactive protein, and DFU history. |
|  |  | (*b*) Report category boundaries when continuous variables were categorized | 8 | To explore whether the variables were dose-dependent on the outcome of amputation of diabetic foot ulcers, we defined the abnormal values of variables according to clinical experience and guidelines[33-37]. Patients were divided based on 11.1-16.7 mmol/L and >16.7 mmol/L blood glucose. Patients were divided based on 25.0-34.9 g/L and < 25.0 g serum albumin levels. Patients were divided based on 8-100 mg/L and > 100 mg/L CRP levels. The duration of diabetes was divided into 0-10 years, 10-20 years and > 20 years. Patients were divided based on creatine levels as follows: 134-186 μmol/L, 187-451 μmol/L, 452-771 μmol/L and > 771 μmol/L. Details are provided in S2 Table. |
|  |  | (*c*) If relevant, consider translating estimates of relative risk into absolute risk for a meaningful time period | Not applicable |  |

Continued on next page

| Other analyses | 17 | Report other analyses done—eg analyses of subgroups and interactions, and sensitivity analyses | 9,10 | Model construction  Statistical analyses were performed using SPSS 26.0. The enumeration data were expressed as count (percentage) and processed with a Chi-square test, whereas the measurement data were presented as the means ± standard deviation and analyzed by t-test. Factors with a p-value <0.05 in the univariate binary logistic regression analysis were used as candidate factors to construct predictive models. In this study, machine learning algorithms were selected as the modeling methods for DFU minor amputation prediction. The whole dataset was randomly divided into the training set and the verification set according to the proportion of approximately 7:3, in which the training set was used to build the prediction models, and the verification set was used to verify and evaluate the performance of the models. Five machine learning algorithms were adopted to build risk prediction models, including DT, RF, LR, SVM and XGBoost. Among them, RF and XGBoost are integrated machine learning classifiers, and the remainder are single classifiers. In the process of training, the optimal parameters were determined by 10 cross-validations. The process of filtering variables and building and evaluating models is shown in Fig 1.  Model evaluation  The evaluation indicators, including accuracy, precision, recall rate, F1-score, and AUC, were calculated to assess the constructed models. The closer the values are to 1, the better the performance of the prediction model. In addition, our experiment also used the receiver operating characteristic curve (ROC) to graphically represent the discernibility. As the most persuasive measurement for predictive analysis in machine learning, we also employed the confusion matrix (CM) for further model evaluation, which is a summarized table of the number of actual values and the predicted values yielded by the prediction model. Details of all these evaluation metrics are shown in Table 1. |
| --- | --- | --- | --- | --- |
| Discussion | | | | |
| Key results | 18 | Summarise key results with reference to study objectives | 18 | To identify the most suitable model for predicting minor amputation in Texas 3 grade DFU patients, our team collected 21 factors based on demographic features, wound features, and laboratory indicators in 362 cases. Five algorithms, namely DT, RF, LR, SVM and XGBoost, were used to predict the minor amputation probability. The XGBoost algorithm performed better than the commonly used linear regression model (LR), the single classifier (DT and SVM), and another ensemble learning machine learning algorithm (RF). Thus, the XGBoost algorithm was best to predict minor amputation of DFU patients (Table 4, Figs 3-4). |
| Limitations | 19 | Discuss limitations of the study, taking into account sources of potential bias or imprecision. Discuss both direction and magnitude of any potential bias | 22, 23 | This study has some limitations. First, this study collected case data from the *** Hospital and the *** Hospital. Because these hospitals serve as the main referral centers for DFU, our conclusions are sufficiently representative. However, the amount of data is limited, limiting the performance of machine learning. We aim to verify the results in a larger sample size of patients from multiple centers in the future. Finally, due to incomplete examination records, we did not include some laboratory indicators that may be related to minor amputation, such as glycosylated hemoglobin and serum insulin levels, or the treatment of other diseases of the patient, such as hypolipidemic agents for ischemic heart disease and anticoagulants for peripheral vascular disease. Assessment of the influence of these indicators as confounding factors can increase the reliability of the predictive model of DFU development.‎ |
| Interpretation | 20 | Give a cautious overall interpretation of results considering objectives, limitations, multiplicity of analyses, results from similar studies, and other relevant evidence | 22 | Our research is characterized by the following points: 1. The study collected data from two tertiary care hospitals in East China, focusing on patients with Texas grade 3 diabetic foot ulcers and the outcome of minor amputations; 2. We compared five popular machine learning algorithms (LR, RF, DT, SVM and XGBoost) to filter the best-performing clinical model. 3. We used the oversampling method (SMOTE) to balance the data; 4. The influencing factors of minor amputation risk were ranked and discussed in depth. |
| Generalisability | 21 | Discuss the generalisability (external validity) of the study results | 22,23 | First, this study collected case data from the *** Hospital and the *** Hospital. Because these hospitals serve as the main referral centers for DFU, our conclusions are sufficiently representative. However, the amount of data is limited, limiting the performance of machine learning. We aim to verify the results in a larger sample size of patients from multiple centers in the future. |
| Other information | |  | | |
| Funding | 22 | Give the source of funding and the role of the funders for the present study and, if applicable, for the original study on which the present article is based | 24 | This work was supported by the National Natural Science Foundation of China (Grant No. 81974288). |

*Give information separately for cases and controls in case-control studies and, if applicable, for exposed and unexposed groups in cohort and cross-sectional studies.

**Note:** An Explanation and Elaboration article discusses each checklist item and gives methodological background and published examples of transparent reporting. The STROBE checklist is best used in conjunction with this article (freely available on the Web sites of PLoS Medicine at http://www.plosmedicine.org/, Annals of Internal Medicine at http://www.annals.org/, and Epidemiology at http://www.epidem.com/). Information on the STROBE Initiative is available at www.strobe-statement.org.
